# The Role of Motivational Regulation in Exam Preparation: Results From a Standardized Diary Study

**DOI:** 10.3389/fpsyg.2019.00081

**Published:** 2019-02-05

**Authors:** Nicole Eckerlein, Anne Roth, Tobias Engelschalk, Gabriele Steuer, Bernhard Schmitz, Markus Dresel

**Affiliations:** ^1^Department of Psychology, University of Augsburg, Augsburg, Germany; ^2^Department of Psychology, Technical University of Darmstadt, Darmstadt, Germany

**Keywords:** self-regulated learning, motivational regulation, quality of strategy use, standardized learning diary, higher education

## Abstract

Previous studies have shown that the use of motivational regulation strategies has the potential to sustain invested effort and persistence in the learning process. Combining different methods (questionnaires and standardized diaries), the present study aimed to determine the role of motivational regulation in an exam preparation period. Motivational regulation is differentiated in a quantitative (extent of strategy use) and a qualitative (planning, implementing, monitoring, and correcting strategy use) aspect. One hundred and fifteen university students reported the quantity and quality of their motivational regulation strategy use in a pretest and kept a standardized learning diary focused on motivational difficulties and invested effort over a 14-day period just before an exam in their studies. Exam performance was assessed afterward. Results revealed positive effects of both aspects of motivational regulation on invested effort in exam preparation and exam performance. Moreover, a high quality of motivational regulation was associated with reduced negative effects of motivational difficulties on invested effort during studying—implying that motivational regulation can buffer against specific motivational problems occurring in the learning process.

## Introduction

Research on motivational regulation has provided a body of evidence which shows its impact on learning behavior, and its importance for the field of self-regulated learning (e.g., Wolters, 2003, 2011; Zimmerman, 2011). Recent studies have shown the relative importance of certain strategies (Schwinger and Otterpohl, 2017). Moreover, research stresses the significance of quality of motivational regulation (Engelschalk et al., 2017), which involves planning, implementing, monitoring and, if necessary, correcting strategy use (e.g., Zimmerman, 2000; Pintrich, 2005). Although increasing numbers of empirical findings signal the importance and functioning of motivational regulation, the results are often based on global measurements of the consequences of motivational regulation. The aim of the present study is to determine the role of motivational regulation in an exam preparation phase using a process-oriented approach. By combining questionnaires and standardized learning diaries, the study aims to provide insights into motivational difficulties and invested effort in the study process and the role of motivational regulation over the course of a 14-day study period in a university setting.

## Motivational Regulation

Self-regulated learning skills are an important determinant of effective learning—this is especially true for environments with large degrees of autonomy, such as higher education (e.g., Streblow and Schiefele, 2006). University students are constantly challenged to plan and implement learning activities and must continuously monitor their own learning processes, intervene if study motivation is too low, and evaluate the outcomes of university learning tasks (Haendel et al., 2013).

Ideal self-regulated learners are able to monitor and regulate their own learning processes by influencing cognitive or metacognitive processes, setting goals, and adjusting behavior to achieve tasks (Winne and Hadwin, 2008). One important aspect of self-regulated learning in this vein is the active control of motivational processes (Garcia and Pintrich, 1994; Boekaerts, 1995, 1997, 1999; Wolters, 1998, 1999; Pintrich, 1999; Sansone and Thoman, 2006). Motivation is an essential factor during all phases of learning: while planning to study, during studying, as well as after studying when learning processes and outcomes are evaluated (Wolters, 2003). Motivational regulation comprises all activities that are used to initiate, maintain, and increase a certain degree of motivation (Wolters, 2003). Self-regulation of motivation can be conceptualized as a process that cyclically repeats itself (Schwinger and Stiensmeier-Pelster, 2012). The learner constantly needs to monitor his or her own motivation and intervene if he or she sees the need for it. Aside from the quantity of strategy use (Schwinger et al., 2009), application quality (Engelschalk et al., 2017) is important for effective motivational regulation. High levels of motivational regulation have shown to be beneficial for learning behavior and learning outcomes, as they ideally lead to increased effort and persistence (Wolters, 2003, 2011; Schwinger et al., 2009; Zimmerman, 2011) and to better achievement (Schwinger and Stiensmeier-Pelster, 2012; Engelschalk et al., 2017).

### Motivational Regulation Strategies

Research has shown that students use a variety of different strategies to regulate their study motivation (Zimmerman and Martinez-Pons, 1986, 1990; Schwinger et al., 2007; Wolters and Benzon, 2013). In the case of motivational regulation, strategies are directed toward influencing one’s own motivational processes. Schwinger et al. (2009) describe eight different motivational regulation strategies: enhancement of situational interest (modifying a tedious learning activity so that it appears to be more interesting), enhancement of personal significance (establishing connections between the learning material and personal interests), mastery self-talk (increasing the goal to study to improve own skills and knowledge), performance-approach self-talk (activating the goal of positive evaluation or good grades), performance-avoidance self-talk (activating the goal of avoiding negative assessment of one’s own performance), self-consequating (setting a reward for reaching a learning goal), proximal goal setting (breaking down a learning goal into smaller immediate goals which appear to be less difficult to attain) and environmental control (minimizing disruptive influences in the learning environment). The use of motivational regulation strategies has been shown to be connected to higher effort and persistence in studying (e.g., Wolters, 1998, 1999, 2003; Schwinger et al., 2009). Schwinger and Stiensmeier-Pelster (2012) classified students according to their quantitative use of different strategies. They found that students with high-intensity strategy-use profiles also reported investing more effort in their course of studies. Wolters and Benzon (2013) found connections between the quantity of strategy use and other aspects of self-regulated learning, such as the use of cognitive and metacognitive strategies, as well as other motivational variables (e.g., value, self-efficacy and goal-orientations). Previous research was not always able to determine significant connections between motivational regulation and achievement, which may not be surprising since the use of motivational regulation strategies is primarily targeted toward increasing motivation (Schwinger et al., 2009). Nonetheless, an indirect effect of motivational regulation on achievement, moderated by effort, is plausible. For example, Wolters (1998) showed that the use of self-consequation and performance-approach self-talk correlated weakly, but positively, with class grades.

### Quality of Motivational Regulation

Motivational regulation strategies can be used with a lower or higher application quality, which means that they are applied more or less accurate, effective, and target-oriented. Thus, it can be argued that it is not only important to use a certain amount of motivational regulation strategies to a certain extent, but the strategies must also be applied properly to ensure that the purpose of regulation is obtained—in this case the goal of regulating one’s own motivation (Engelschalk et al., 2017).

Target-aimed self-regulation requires an accurate, coordinated and controlled realization of motivational regulation strategies (Zimmerman, 2000; Pintrich, 2005; Winne and Hadwin, 2012). It can be assumed that effective motivational regulation involves a series of metacognitive processes such as planning, implementing, monitoring and correcting strategy use (e.g., Zimmerman, 2000; Pintrich, 2005). The importance of quality of motivational regulation can be examined in the following example (cf., Engelschalk et al., 2017): A student might be preparing for an exam and realize that her motivation to continue studying is too low. After determining to regulate her motivational difficulties, she decides to set certain sub-goals for the rest of day. She actively plans the use of this motivational regulation strategy and implements the strategy with a high degree of quality, meaning that the sub-goals will divide the goal for the day into reasonably sized units. While studying, she monitors strategy use and constantly evaluates whether the strategy is implemented correctly and helps to improve her study motivation. If she realizes that the strategy does not improve her motivation, the next move should be to adjust the strategy used or to switch to another strategy. These metacognitive control processes ensure a high quality of self-regulation (see Winne and Hadwin, 2008). Consequently, motivational regulation strategies are assumed to be particularly effective if they are implemented, monitored, and adapted throughout the learning process. Thus, if the student in the example implements the strategy without monitoring strategy use, she might not be able to increase her study motivation, which will increase her risk to give up on studying for the day.

Engelschalk et al. (2017) analyzed the importance of both the quantity and the quality of motivational regulation for study effort and achievement in undergraduate students. The authors found that effort and achievement were significantly better predicted by including the quality of motivational regulation aside from its quantity. In an earlier study, Leutner et al. (2001) trained students to use the motivational regulation strategy enhancement of personal significance. One experimental group of students was additionally trained with regard to application quality, by being asked to monitor and reflect strategy use, reflect if the strategy was useful in increasing motivation and adapting strategy use if necessary. The authors could show that the students who also received the application quality training reported a higher degree of study motivation and received better grades on a final test (Leutner et al., 2001). Although only few studies yet have targeted the quality of motivational regulation, the findings indicate that quality of motivational regulation has additional value in predicting proximal aspects and distal outcomes of learning processes.

### Assessing the Process of Self-Regulated Learning

While studying for an exam or working on an academic paper, students repeatedly deal with motivational difficulties and obstacles (e.g., Klug et al., 2011). Moreover, motivational regulation and self-regulation are, in general, cyclical processes that pose a variety of complex demands on the learner (Winne and Hadwin, 2008). To capture the process of self-regulated learning over time and to permit observations of changes and obstacles that might occur along the way, we argue to focus on process-oriented and situation-specific measurements (see Roth et al., 2016). Learning diaries allow for an assessment of self-regulated behavior over a series of measuring points (Schmitz and Perels, 2011), and have already been successfully used in process-oriented self-regulated learning research (e.g., Schmitz and Wiese, 2006; Stoeger and Ziegler, 2008).

The present study utilizes a standardized learning diary approach that allows the capturing of motivational difficulties and invested effort in the learning process. The learning diary allows a situation-specific assessment of those variables. Thus, the students do not need to generalize across the entire learning process and the learning diary also allows us to gain insight into varying study situations and trajectories of individual students over a specific period of time (Schmidt et al., 2011). Assessing self-regulation behavior over multiple measuring points is a reliable and valid method that can depict tendencies and variations occurring during the learning process (Schmitz and Perels, 2011). A particular advantage of learning diaries is their high ecological validity, as these instruments capture the learning process itself. Kanfer et al. (1999) correlated self-reported diary data in clinical studies with data obtained by external observers and could confirm high accuracy and reliability for learning diary data. Schiefele (2005) used a learning diary approach to show that strategy use changes during the different phases of preparing for an exam. The process data showed that students used different strategies in different phases of the learning process, and that the usage pattern could predict exam grades. Additionally, Boekaerts and Corno (2005) suggest that students are more open to learning diaries than to other forms of assessment, e.g., standardized questionnaires.

### Aims and Hypotheses

While preparing for an exam, students might need to cope with situations involving motivational problems, struggle with daily fluctuations in their learning motivation, and face the task of regulating their own motivation to start or maintain learning behavior. The central focus of this paper is the examination of the role of motivational regulation over time during the specific phase of preparing for an exam and its motivational problems, and the effects quantity and quality of motivational regulation have on this process. In addition to the quantity of motivational regulation, regulation quality was included in the present work. We assume a combination of quantity and quality of motivational regulation to be a precondition of effective motivational regulation in the learning process. Particularly, the following hypotheses were formulated:

Hypothesis 1: Quantity and quality of motivational regulation are positively connected to proximal (invested effort) and distal (achievement) outcomes of the learning process.
Hypothesis 2: Quantity and quality of motivational regulation moderate (i.e., weaken) the negative effects that motivational difficulties, experienced while studying, have on the effort invested in the learning process.

It is expected that students who use motivational regulation strategies in a larger quantity and with a high degree of quality should show higher rates of invested effort, even when facing motivational difficulties. In contrast, students who report using very few strategies, and regulate their motivation with a low quality, should show lower rates of invested effort when facing motivational difficulties, because they are not able to cope with these difficulties. Consequently, we expect that students with a high quantity and quality of motivational regulation show higher rates of invested effort in the exam preparation phase in general and, eventually, better grades on their final exams.

We expected that all predicted associations would hold true even after controlling for potential dependencies of prior achievement.

## Materials and Methods

### Participants

The sample consisted of 115 students enrolled at two mid-sized German universities. Their average age was 23.9 years (*SD* = 4.7) and 74.8% were female. On average, the students were in their fifth semester (*M* = 5.2; *SD* = 3.8). Of these students, 46.1% were enrolled in psychology and 53.9% in educational studies. Students participated voluntarily. For participation, they received a small amount of money and entered a lottery, where prices could be won for filling in the diary regularly.

The study was conducted in full accordance with the Ethical Guidelines of the American Psychological Association. At the time the data was acquired, it was not customary at most German universities to seek ethics approval for survey studies on self-regulated learning. No identifying information was acquired from participants, as the study made use of anonymous questionnaires and data was matched solely relying on codenames.

### Procedure and Instruments

The underlying design of the study was longitudinal. The learning diary was administered online. The students were instructed to maintain the learning diary for 14 days leading up to a psychology exam at the end of a semester, which allowed us to track changes in perceived motivational difficulties and invested effort over each day. The pretest was conducted 2 weeks prior to the exam preparation period. Follow-up data was collected 6 months after the exam (this long period was necessary since the exam within educational studies had a long correction time). The pretest and follow-up both took the form of an online questionnaire. The learning diary was designed following Schmitz and Perels (2011) and assessed motivational difficulties and invested effort after studying. The students filled in the standardized diary, respectively, for studying for the psychology exam. In a standardized part, the journal assessed self-reported motivational difficulties that occurred while studying, and the self-reported amount of effort invested each day. Other constructs were measured in the learning diary (e.g., subjective well-being after studying), which are not addressed in the present analyses. An average of 10.5 (*SD* = 3.0) entries were made per student over the 14-day exam preparation period. Altogether 1,208 data entries were used in the analyses. Students who had fewer than three entries were excluded from the study due to inadequate response, resulting in a minimum of three and a maximum of 14 entries per student.

The extent of current *motivational difficulties* in the learning process was assessed in the standardized learning diary with the item “Today I struggled to keep my study motivation on a high level” on a Likert scale from 1 (*don’t agree at all*) to 6 (*totally agree*).

Daily *invested effort* was assessed in the standardized learning diary with the item “I tried especially hard today” on a Likert scale from 1 (*don’t agree at all*) to 6 (*totally agree*).

*Quantity of motivational regulation* was assessed in the pretest with an instrument developed by Schwinger et al. (2007). Its validity has been proven in many studies (Schwinger et al., 2009; Grunschel et al., 2016; Schwinger and Otterpohl, 2017). Students had to rate the quantity of strategy use in general with 30 items, for the eight different motivational regulation strategies mentioned above, on a Likert-type scale ranging from 1 (*rarely/never*) to 5 (*very often*). One example item reads: “I look for connections between the tasks and my life as such” (enhancement of personal significance). The subscale of performance-avoidance self-talk was excluded from the motivational strategies index because it is frequently regarded as a maladaptive strategy (e.g., Schwinger and Otterpohl, 2017). All items were compiled into a single score (motivational strategies index; Schwinger et al., 2009) indicating quantity of strategy use (α = 0.68).

*Quality of motivational regulation* was assessed situation-specifically in the pretest with a total of 12 items in four standardized situational vignettes (for a full description of the instrument and its validity see Engelschalk et al., 2017). The four situational vignettes represented different prototypical motivational problems and were defined on the basis that learners distinguish between motivational problems that stem from a low expectancy for success or a low subjective value for the subject or outcome of the learning task, either before starting to study or while studying (Engelschalk et al., 2016). These four combinations of motivational problems were assessed with reference to the learning situation of preparing for an exam. Students then had to state which strategy they would use in the given situation to regulate their own motivation in an open-ended format. Two examples of stated strategies are: “I plan an activity with my friends in the evening to reward myself for revising the script” and “I think about the importance of the subject for my future job.” Subsequently, three aspects of quality of motivational regulation were assessed with one item each answered on a Likert-type scale ranging from 1 (*don’t agree at all*) to 6 (*totally agree*): target orientation (“I apply this strategy in a manner that will effectively improve my motivation”), accuracy (“In the application of this strategy, I am very precise”), and control (“When I use this strategy, I check regularly to determine if my motivation is improving or not”). The items of all situations were compiled to one single score indicating the overall quality of strategy use. The internal consistency was sufficient (Cronbach’s α = 0.75).

*Achievement* data was assessed by means of self-reported marks the students received on their psychology exams in a posttest conducted 6 months after the exams (due to long correction cycles involving two independent auditors, the notifications of the grades were usually in close proximity to this assessment). Thus, the achievement data is directly related to the exam preparation phase for which the students used the learning diaries. The responses, which were based on the German reversed grading system, were transformed to a scale ranging from 1 (indicating *insufficient performance*) to 15 (indicating *excellent performance*) to improve the interpretability of the results.

*Previous achievement* was included as a control variable. The high school diploma grade (similar to the grade point average that is used, for example, in the U.S. American system) was assessed in the pretest and transformed to a scale ranging from 1 (*insufficient performance*) to 15 (*excellent performance*).

### Dealing With Missing Data

Of the study participants, 47.2% did not supply complete information on their exam grade in the follow-up study. In order to exclude potential biases in the results due to non-random missing data in this variable, we compared students who did not report their exam grades with students who provided this information with regard to all other variables using *t*-tests for independent samples. These additional analyses revealed no systematic differences between the two groups [*t*(113) < 0.89; *p* > 0.05] indicating that the results of the study are not affected by the relatively large missing rates in the follow-up. For all variables other than achievement, missing data due to item non-response was quite infrequent and not higher than 2%. Missing data was imputed using the expectation-maximization algorithm (Peugh and Enders, 2004).

### Analyses

The longitudinal diary data was analyzed using hierarchical linear modeling (Singer and Willet, 2003). This allows a process-oriented analysis of motivational difficulties, invested effort and their relations in their temporal variability and to analyze the influences of inter-individual differences in motivational regulation on these aspects. A two-level model was used, in which measuring points (single diary entries) are clustered within persons. All models were estimated using HLM 6.06 (Raudenbush et al., 2004). All variables were *z*-standardized prior to analysis (motivational difficulties and invested effort over all measuring points) to allow for an interpretation of the estimated coefficients analogously to standardized regression coefficients.

Two models were estimated. To estimate the proportion of between-person variation (constant variation between persons) and within-person variation (change over time within persons), an unconditional means model (Model 1) was estimated for the outcome variable EFF_ij_ (invested effort) observed for person_i_ at measuring point_j_:

$$\begin{array}{r} \text{Level }1(\text{measuring points}):{\text{EFF}}_{\text{ij}}={\text{π}}_{\text{0i}}+{\text{e}}_{\text{ij}}\\ \text{Level }2(\text{persons}):{\text{π}}_{\text{0i}}={\text{β}}_{\text{00}}+{\text{r}}_{\text{0i}} \end{array}$$

The outcome variable EFF_ij_ to a given point in time is expressed within persons as the sum of a person-specific mean π_0i_ over all measuring points and a measuring point-specific residuum e_ij_ from this average. Between persons, the person-specific mean π_0i_ is expressed as the sum of the sample mean β_00_ of these person-specific parameters and a person-specific residuum r_0i_.

To determine the effects of motivational regulation (quantity and quality) as between-person factors on invested effort (Hypothesis 1) and the relationship between motivational difficulties and invested effort (Hypothesis 2), the model was extended to include motivational difficulties (DIF_ij_), quantity of motivational regulation (MRQUAN_i_) and quality of motivational regulation (MRQUAL_i_) (Model 2). Motivational difficulties were inserted as time-varying predictor with a random slope in order to allow for inter-individual differences in the effect of these difficulties on invested effort. Quantity and quality of regulation were included twice, namely (a) as predictors of the person-specific intercept (π_0i_) to test their effects on the level of invested effect and (b) as predictors of the person-specific slope (π_1i_) of motivational difficulties to test motivational regulation’s expected capability to reduce the negative effects of these difficulties. To control for potential systematic changes in time (e.g., generally intensified effort shortly before for the exam), time in days was inserted additionally in Model 2.

Level 1 (measuring points):

$${\text{EFF}}_{\text{ij}}={\text{π}}_{\text{0i}}+{\text{π}}_{\text{1i}}\;\cdot {\text{DIF}}_{\text{ij}}+{\text{π}}_{\text{2i}}{\text{TIME}}_{\text{ij}}+{\text{e}}_{\text{ij}}$$

Level 2 (persons):

$$\begin{array}{l} {\text{π}}_{\text{0i}}={\text{β}}_{\text{00}}+{\text{β}}_{\text{01}}{}_{}\cdot {\text{MRQUAN}}_{\text{i}}+{\text{β}}_{\text{02}}\cdot {\text{MRQUAL}}_{\text{i}}+{\text{r}}_{\text{0i}}\\ {\text{π}}_{1\text{i}}={\text{β}}_{\text{10}}+{\text{β}}_{\text{11}}{}_{}\cdot {\text{MRQUAN}}_{\text{i}}+{\text{β}}_{\text{12}}\cdot {\text{MRQUAL}}_{\text{i}}+{\text{r}}_{\text{1i}}\\ {\text{π}}_{\text{2i}}={\text{β}}_{\text{20}}+{\text{r}}_{\text{2i}} \end{array}$$

To determine relations between quantity and quality of motivational regulation and exam achievement as distal outcome of the learning process, bivariate correlations were calculated additionally.

## Results

Table 1 shows means, standard deviations, and bivariate correlations for all variables.

**Table 1:** Descriptive statistics and bivariate correlations.

			Range						
	*M*	*SD*	Potential	Actual	2	3	4	5	6
1. Quantity of motivational regulation	3.2	0.48	1–5	2.5–4.8	0.49^∗∗^	-0.25^∗∗^	0.39^∗∗^	0.38^∗∗^	–0.02
2. Quality of motivational regulation	4.3	0.74	1–6	2.5–5.8		–0.28^∗∗^	0.47^∗∗^	0.33^∗∗^	–0.00
3. Motivational difficulties ^a^	3.6	0.70	1–6	1.6–5.1			–0.47^∗∗^	–0.08	–0.11
4. Invested effort ^a^	4.3	0.65	1–6	2.3–5.4				0.19^∗^	–0.04
5. Achievement (exam grade)	6.1	2.30	1–15	1–10					0.25^∗∗^
6. Previous achievement (high school diploma)	11.7	1.70	1–15	8–15					

*N = 115 university students. ^a^Bivariate correlations were calculated using aggregated scores over all measuring points in the learning diary. ^∗∗^p < 0.01, ^∗^p < 0.05.*

### Hierarchical Linear Modeling of Invested Effort in Dependence of Motivational Difficulties and Motivational Regulation

To differentiate between-person variations independent of time and within-person variations over time, the unconditional means model (Model 1) was estimated (Table 2). The variance components observed indicate substantial between-person variation (*ICC* = 0.18).^1^ Though, more important in the context of this study is the quite large within-person variation, which was much larger than inter-individual differences. Thus, effort invested in the learning process is strongly characterized by variations with the learning situation rather than being a stable person-specific variable.

**Table 2:** Hierarchic linear prediction of invested effort by motivational difficulties and motivational regulation.

Parameter	Model 1	Model 2
**Fixed effects**		
Intercept β_00_	-0.02 (0.05)	0.00 (0.04)
Quantity of motivational regulation β_01_		0.09^*^ (0.04)
Quality of motivational regulation β_02_		0.17^***^ (0.04)
Motivational difficulties β_10_		-0.45^***^ (0.03)
Quantity of motivational regulation β_11_		0.01 (0.03)
Quality of motivational regulation β_12_		0.07^*^ (0.03)
Time in days β_20_		0.06^***^ (0.01)
**Random parameters**		
*E* = *Var*(*e_ij_*)	0.83	0.56
*R*_0_ = *Var*(*r*_0_*_i_*)	0.18^***^	0.10^***^
*R*_1_ = *Var*(*r*_1_*_i_*)		0.05^***^

*N = 115 students with altogether 1,208 diary entries. All variables were z-standardized prior to analysis. Standard errors are in parentheses. E = Var(e_ij_) estimates the within-person variance of the outcome; R_0_ = Var(r_0__i_) estimates the between-person variance. R_1_ = Var(r_1__i_) estimates the variance between persons in the individual growth rate. ^∗∗∗^p < 0.001, ^∗^p < 0.05.*

To explain time-specific invested effort and its interplay with motivational difficulties in dependence of motivational regulation, Model 2 was estimated (see Table 2). Aside from an average increase in invested effort over time toward the exam date, the results indicated general positive effects of motivational regulation on invested effort in the first instance as indicated by significant coefficients β_01_ and β_02_ (Hypothesis 1). The larger the extent of using motivational regulation strategies and the better the quality of this strategy use (as reported by the students in the pretest), the larger was the level of invested effort within the phase of preparing for the exam. Descriptively, the effect of quality of motivational regulation seems to be somewhat stronger than the effect of regulation quantity.

Concerning motivation difficulties that may frequently appear in learning processes, it was assumed that motivational regulation moderates (i.e., reduces) their negative impact on effort invested in the learning process (Hypothesis 2). The analyses show that motivational difficulties had, on average, a significant and relatively strong negative effect on invested effort (β_10_)—indicating that students invest lower levels of effort when they are faced with motivational problems during studying.

**FIGURE 1:**
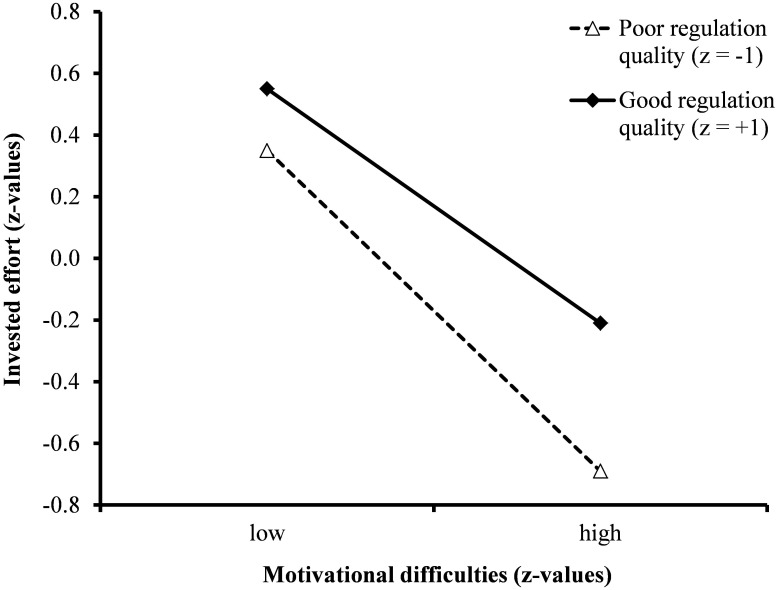
Moderating effect of regulation quality on the connection between motivational difficulties and invested effort (depicted are predicted values).

Relevant for testing Hypothesis 2 is the cross-level interaction effect between motivational difficulties and motivational regulation (β_11_ and β_12_). Here, quality of motivational regulation moderated the negative effect of motivational difficulties on invested effort. In other words, students with a high quality of motivational regulation showed higher levels of invested effort even if they are confronted with motivational difficulties. On the other hand, students with poor regulation quality were especially vulnerable for experiencing motivational difficulties during learning. The moderating effect of quality of strategy use on the connection between motivational difficulties and invested effort is depicted in Figure 1. A similar protecting effect was not found for the quantity aspect of motivational regulation.

To control for potential dependencies of the found relationships on previous achievement, the hierarchical linear analyses were repeated including the high school diploma grade as predictor for both, the intercept and the slope of motivational difficulties. All found effects were robust, i.e., did not change substantially in their size and stayed statistically significant.

### Motivational Regulation and Achievement

Positive bivariate correlations between both aspects of motivational regulation assessed in the pretest and exam grade assessed in the follow-up were observed (see Table 1). Accordingly, students with a higher quantity of motivational regulation and a better quality of strategy use reported significantly better grades on the final exam.

Additionally, multiple regression analysis was conducted to simultaneously estimate the effects of both, quantity and quality of motivational regulation on achievement. The results are reported in Table 3. As expected, quantity as well as quality of motivational regulation proved to be moderately positive predictors of achievement in the final exam.

**Table 3:** Regression of achievement (exam grade) on quantity and quality of motivational regulation.

	Achievement
Quantity of motivational regulation	0.29^**^
Quality of motivational regulation	0.18^*^
*R*^2^	0.17

*N = 115 university students. Standardized regression coefficients are depicted. ^∗∗^p < 0.01, ^∗^p < 0.05.*

The regression analysis was repeated with previous achievement as a control measure—in order to rule out concerns that the effects of motivational regulation on achievement simply reflect that students with better prior performances are also better able to regulate their motivation while studying. Again, the effects were robust—previous achievement predicted achievement in the pertaining exam, but did not diminish the contributions of quantity and quality of motivational regulation on exam performance. Thus, quantity and quality of motivational regulation predicted achievement above and beyond the effects of previous achievement—which is in line with the assumption that motivational regulation has a causal effect on achievement.

## Discussion

The present study was designed to examine the role of quantity and quality of motivational regulation when it comes to motivational difficulties in the process of exam preparation. The use of a standardized learning diary approach provided unique insight into the everyday process of studying. This approach allowed us to capture impending motivational difficulties in the process, and to reconstruct the daily ups and downs of a study period. In contrast to a solely global assessment of self-regulated learning, the students did not have to generalize and abstract their motivational problems and invested effort from many situations to a global level. Another strength of the present work is the incorporation of both the quantity and quality of motivational regulation.

The findings indicate that motivational difficulties as triggers for motivational regulation are indeed situation-specific and can fluctuate strongly from day to day, rather than being a constant person-specific variable. The large proportions of within-person variance indicated that university students frequently struggle with keeping invested effort high while encountering motivational difficulties when preparing for an exam. The results confirmed that motivational difficulties lead, on average, to lower rates of invested effort in the learning process, which endangers study success.

Notably, the present study shows that a high quality of motivational regulation is associated, as expected in Hypothesis 2, with a weaker negative effect of motivational difficulties on invested effort. Thus, students who plan, monitor, and adapt (when necessary) their use of motivational strategies are obviously more effective in regulating their motivational difficulties (Zimmerman, 2000; Pintrich, 2005; Engelschalk et al., 2017). Thus, the negative impact of motivational difficulties tends to be less severe for students who can regulate their motivation with a high degree of quality while other students are more vulnerable to motivational difficulties. Consequently, the application of high quality strategies can protect study effort against motivational struggles that occur while studying. Independent from motivational difficulties, students with higher quantity and quality of motivational regulation generally showed higher levels of invested effort during the learning process and, notably, reported better grades in their final exams (Hypothesis 1). These results are in line with the assumption that both a high quantity and a good quality of motivational regulation foster learning and achievement. Previous work in the field frequently postulated this effect, but was seldom able to prove it in an unconditioned fashion. The connection between motivational regulation and achievement was found to be moderated by intelligence (Schwinger et al., 2009), to be valid only for selected motivational regulation strategies (Wolters, 1998), only for a high quality of motivational regulation (Engelschalk et al., 2017), or not at all. The present work can therefore be interpreted as evidence for the relevance of the motivational regulation concept not only for improving one’s motivation but also for eventually improving one’s achievement. The findings align with research that has shown that the regulation of one’s own motivation is a demanding task that has proven to be crucial for proximal and distal learning outcomes (e.g., Leutner et al., 2001; Engelschalk et al., 2017; Schwinger and Otterpohl, 2017).

In the present study, quantity and quality of motivational regulation were associated with achievement, but only a high quality of motivational regulation was able to moderate the negative effects of motivational difficulties on invested effort in the specific situations of the learning process. From a theoretical point of view this result pattern is sensible—only students who monitor and adapt the application of motivation regulation strategies should be able to overcome motivational difficulties.

It is important to note that all found effects were robust also after controlling for previous achievement. Consequently, concerns can be ruled out that the found relationships may be due to common correlations with prior achievement or simply reflect causal effects from achievement on motivational regulation. Instead, they indicate that quantity and quality of motivational regulation have an incremental effect on effort and achievement above and beyond the effects of previous achievement.

The present study is subject to certain limitations. Motivational regulation is captured via two core aspects: quantity and quality of motivational regulation. Taking these two elements of motivational regulation into account is, as mentioned above, a strength of the present work. Nevertheless, the influence of another core factor—the aspect of fit between the problematic situation and the strategy at hand—could not be evaluated (cf., Engelschalk et al., 2016). In subsequent studies, all three aspects of motivational regulation (quantity, quality, and situation-specific fit of strategy use) should be examined in a way that allows for a separate analysis of all aspects and their interaction. Moreover, it would also be desirable to develop process-oriented instruments to assess all key aspects of motivational regulation in the learning process itself—although the present study made an important step forward in analyzing motivational regulation in the concrete learning situation. Additionally, the relatively high rate of missing data for the exam grade should be mentioned. However, as missing values occurred independently from all other assessed variables, it is unlikely that the results are biased by them. Finally, it has to be considered that all analyses are based on self-reported data. This approach has been criticized because it may not be a valid measure of self-regulated learning (e.g., Winne, 2010). It would be interesting to align the findings of the present study with data from observational studies and to include behavioral measures for actual strategy use in the learning process. One approach could be a software similar to gStudy (Winne et al., 2006), which allows tracing students’ study methods, adapted to the context of motivational regulation, or utilizing thinking aloud protocols—which, nevertheless, have their own diagnostic limitations (Roth et al., 2016).

Despite these limitations, it can be concluded that motivational regulation is a relevant and demanding aspect of self-regulated learning. The findings point out that motivational difficulties can endanger learning success on a daily basis, as they result in lower rates of invested effort. The results of this study indicate that quality of motivational regulation is an important buffer and protective factor in this process. The findings are in line with the theoretical assumption, that both a high quantity and a high quality of motivational regulation enable students to cope with motivational difficulties, to show high levels of invested effort despite such difficulties and, eventually, to improve their academic performance. The findings, alongside other studies in the field (e.g., Leutner et al., 2001), indicate that not only the quantitative aspect, but also regulation quality is an important aspect of motivational regulation that should be in focus when assessing and training motivational regulation. While choosing a strategy often depends on personal preferences and the individual learning history, all students can profit from improvements to the metacognitive control of their strategy implementation.

## Author Contributions

NE conducted the analyses and wrote the manuscript with support from AR, TE, GS, BS, and MD.

## Conflict of Interest Statement

The authors declare that the research was conducted in the absence of any commercial or financial relationships that could be construed as a potential conflict of interest.

## Footnotes

1 The unconditional means model for motivational difficulties showed similar between-person variation (*ICC* = 0.12).
